# Predictive model and risk analysis for peripheral vascular disease in type 2 diabetes mellitus patients using machine learning and shapley additive explanation

**DOI:** 10.3389/fendo.2024.1320335

**Published:** 2024-02-28

**Authors:** Lianhua Liu, Bo Bi, Li Cao, Mei Gui, Feng Ju

**Affiliations:** ^1^ International School of Public Health and One Health, Hainan Medical University, Haikou, Hainan, China; ^2^ Department of Endocrinology, Second Affiliated Hospital of Hainan Medical University, Haikou, Hainan, China

**Keywords:** type 2 diabetes mellitus, peripheral vascular disease, predictive model, risk factor, machine learning, shapley additive explanation

## Abstract

**Background:**

Peripheral vascular disease (PVD) is a common complication in patients with type 2 diabetes mellitus (T2DM). Early detection or prediction the risk of developing PVD is important for clinical decision-making.

**Purpose:**

This study aims to establish and validate PVD risk prediction models and perform risk factor analysis for PVD in patients with T2DM using machine learning and Shapley Additive Explanation(SHAP) based on electronic health records.

**Methods:**

We retrospectively analyzed the data from 4,372 inpatients with diabetes in a hospital between January 1, 2021, and March 28, 2023. The data comprised demographic characteristics, discharge diagnoses and biochemical index test results. After data preprocessing and feature selection using Recursive Feature Elimination(RFE), the dataset was split into training and testing sets at a ratio of 8:2, with the Synthetic Minority Over-sampling Technique(SMOTE) employed to balance the training set. Six machine learning(ML) algorithms, including decision tree (DT), logistic regression (LR), random forest (RF), support vector machine(SVM),extreme gradient boosting (XGBoost) and Adaptive Boosting(AdaBoost) were applied to construct PVD prediction models. A grid search with 10-fold cross-validation was conducted to optimize the hyperparameters. Metrics such as accuracy, precision, recall, F1-score, G-mean, and the area under the receiver operating characteristic curve (AUC) assessed the models’ effectiveness. The SHAP method interpreted the best-performing model.

**Results:**

RFE identified the optimal 12 predictors. The XGBoost model outperformed other five ML models, with an AUC of 0.945, G-mean of 0.843, accuracy of 0.890, precision of 0.930, recall of 0.927, and F1-score of 0.928. The feature importance of ML models and SHAP results indicated that Hemoglobin (Hb), age, total bile acids (TBA) and lipoprotein(a)(LP-a) are the top four important risk factors for PVD in T2DM.

**Conclusion:**

The machine learning approach successfully developed a PVD risk prediction model with good performance. The model identified the factors associated with PVD and offered physicians an intuitive understanding on the impact of key features in the model.

## 1 Introduction

Diabetes is a rapidly escalating global health emergency of the 21st century. The number of people with diabetes is projected to rise from 536.6 million adults (20-79 years) (10.5% global prevalence) in 2021 to 783.2 million (12.2% global prevalence) in 2045. Over 3 in 4 people with diabetes live in low- and middle-income countries. China is expected to have 174.4 million people with diabetes by 2045, ranking first in the world (1). Type 2 diabetes mellitus(T2DM) is the most prevalent form, accounting for nearly 90% of all diabetes worldwide (2). Complications from diabetes are widespread, with over 50% of individuals with T2DM experiencing microvascular complications and 25% suffering from macrovascular complications (3). These complications not only affect patients’ quality of life and can even lead to death (4–7),but also impose a significant economic burden on society (8–10).

Peripheral vascular disease (PVD) is considered as one of the major macrovascular complications in T2DM due to its wide range of clinical features and consequences (11). It is primarily caused by arterial atherosclerosis and increased blood viscosity, resulting in vascular stenosis or occlusion in the lower limbs. Severe cases may result in foot ulcers, ischemic necrosis, and various other complications, leading to a poor long-term prognosis (12, 13). As a common complication, PVD affects over 10%-20% of individuals with diabetes (14), and approximately 50% in developed countries. In high-income countries, individuals with diabetes now develop PVD as their initial vascular disease (15). Up to 50% of patients with diabetic foot ulcers suffer from peripheral artery disease, and it is an independent risk factor for its development (16). Additionally, PVD is a major cause of amputation and also increases the risk of stroke and death (17). Given the numerous adverse effects of PVD on diabetic patients, early diagnosis and treatment are very crucial to manage it effectively and prevent progression to more severe health issues. However, due to the complex pathogenesis of PVD and often asymptomatic in the early stages (14), there is a high risk of misdiagnosis. Therefore, developing PVD risk prediction models and analyzing the influencing factors for individuals with diabetes is essential to assist clinicians in early prevention and treatment.

With the rapid development of artificial intelligence, machine learning(ML) algorithms are commonly used to build risk predictive models in medicine, as they offer a powerful tool for analyzing large amounts of clinical data and identifying patterns and trends behind the data, which help healthcare professionals better understand the factors that contribute to various diseases (18, 19). In recent years, multiple ML-based prediction models have been developed for assessing the risk of diabetes complications, such as diabetes-related kidney disease (20–23), diabetic retinopathy (24–27) and diabetic peripheral neuropathy (28, 29). However, to date, no ML-based risk prediction model has been specifically developed for PVD in patients with T2DM.

The aim of this study was to develop and evaluate prediction models specifically for PVD in T2DM, as well as to identify risk factors. The most effective model was selected for further analysis using the SHAP method to improve the clinical applicability of the models and ultimately to improve patient outcomes.

## 2 Materials and methods

**Figure 1:**
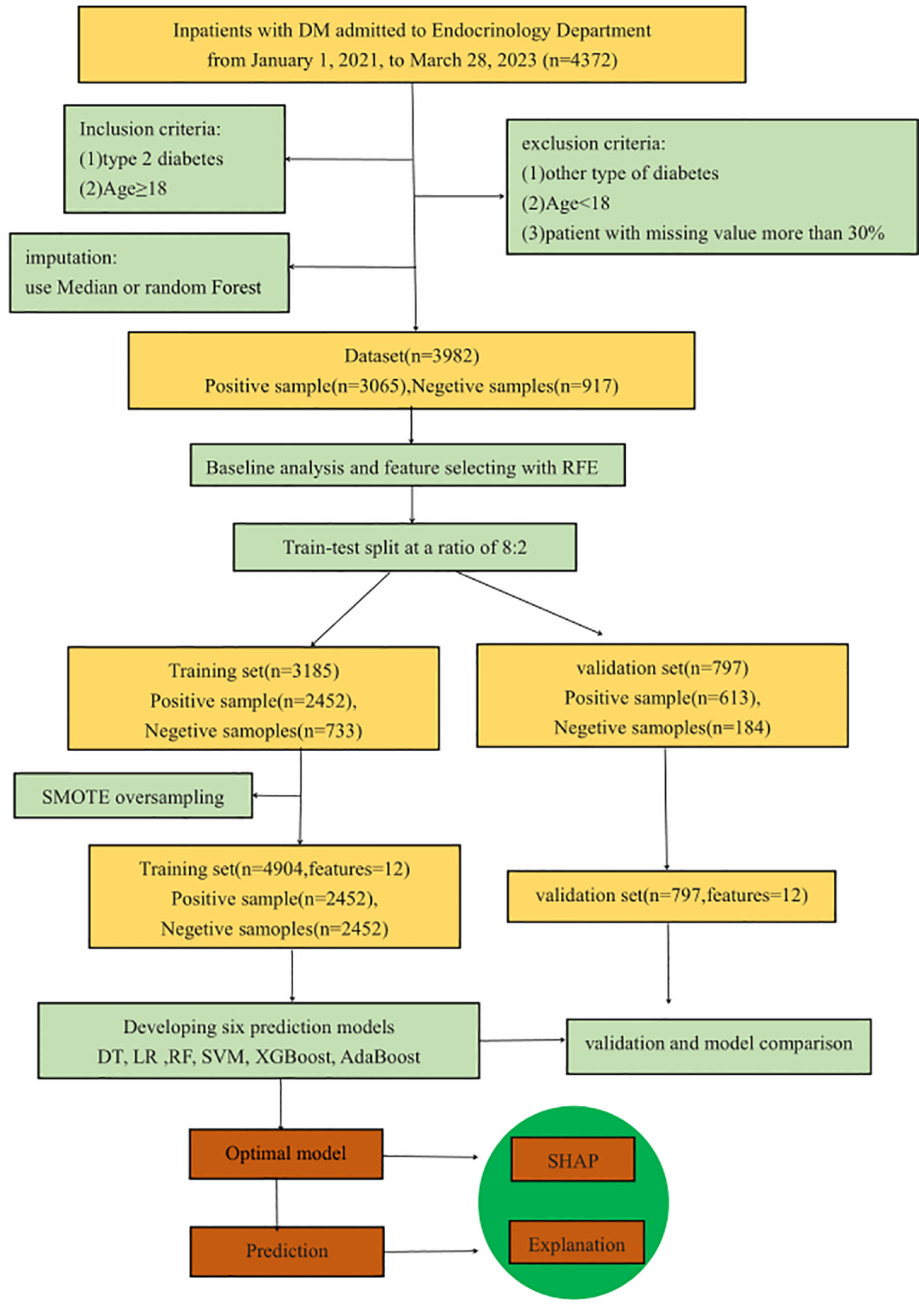
Work flow for prediction model developing, evaluation and interpretation.

In this retrospective modeling study, a three-step process was utilized for model development, validation, and interpretation. Initially, models were developed using six ML methods. Subsequently, model performance was assessed using an internal validation dataset. Finally, the optimal model was interpreted using the SHAP method. **Figure 1** depicts the entire research process, including inclusion and exclusion criteria, feature selection, dataset division, data balancing, model development and validation, model comparison, and optimal model selection and interpretation. The preprocessing of the data, the implementation of the ML models, and the model interpretation were implemented in Python 3.9 by using scikit-learn 1.2.2.

### 2.1 Data collection and candidate predictors

The study was approved by the Ethics Committee of Hainan Medical University(HYLL-2021-388). It was a retrospective study. Data were sourced from the Electronic Health Medical Record System of the Second Affiliated Hospital of Hainan Medical University. Included were hospitalized diabetic patients admitted to the Endocrinology Department between January 1, 2021, and March 28, 2023, comprising total of 4,372 inpatients records. The data consists of three parts: demographic information of 4,372 patients, discharge diagnosis information, and 948,900 laboratory test results. Informed consent was waived as all data were de-identified. The diagnosis of PVD was made according to current guidelines and ancillary tests, and the tests were primarily based on the use of color Doppler ultrasound to observe the presence or absence of atherosclerotic plaques. Inclusion criteria were: (1) confirmed T2DM patients; (2) aged ≥18 years. Exclusion criteria included: (1) other types of diabetes; (2) aged<18 years. Applying these criteria, 4,070 T2DM patients were selected for the study.

The original dataset contained over 300 variables. We selected 67 potential predictors that could influence PVD progression based on current relevant research (4, 30–32) and clinician recommendations. The selected variables included: demographic and clinical characteristics (7 variables), such as age, weight, height, body mass index (BMI), diastolic blood pressure (DBP), systolic blood pressure (SBP), and blood pressure difference (BP diff); discharge diagnosis characteristics (15 comorbidities), including hypertension, diabetic retinopathy (DR), diabetic nephropathy (DN), diabetic peripheral neuropathy (PND), and other related complications; biochemical characteristics (45 variables), including hemoglobin (Hb), glycated Hemoglobin (HbA1c), lipoprotein (a) (LP-a), total bile acids (TBA), total cholesterol (TC), triglycerides (TG), high-density lipoprotein(HDL), low-density lipoprotein (LDL), cystatin c (CysC), alkaline phosphatase (ALP), gamma-glutamyl transpeptidase (GGT), aspartate aminotransferase (AST), alanine transaminase (ALT), total protein (TP), total bilirubin (TBIL), albumin (ALB), blood urea nitrogen (BUN), creatinine (Cr), and so on.

### 2.2 Data pre-processing

Data preparation is crucial in big data applications. We first extracted complication information from the discharge diagnosis and then extracted the biochemical indicators from 948,900 laboratory tests. For variables with multiple measurements, values nearest to the discharge time were extracted. Indicators with over 25% missing data were eliminated, except for retained C-reactive protein, Hb, and TBA, due to their relevance to PVD. Samples with over 30% missing values were excluded. The study ultimately included 3,982 samples, with 3,065 cases of PVD and 917 without. Indicators with missing values under 30% were filled using the median for each category. For values with missing data over 30%, were filled with Random Forest (33), which imputes missing values using random forests in an iterative fashion (34). Considering most of the variables in the dataset are numerical and the range of different variables widely varied. To ensure model accuracy, we standardized the training set using a normalization and applied the same scaling to the test set.

### 2.3 Statistical analysis

All statistical analyses were conducted using IBM SPSS Statistics version 22.0. The Kolmogorov-Smirnov (K-S) method tested the normality of continuous variables, expressed as mean± SD or median (P25, P75) for skewed distributions. Categorical variables were described as n (%). Comparisons between the PVD and non-PVD (NPVD) groups utilized Student’s T-test, Mann-Whitney U test, or Chi-squared test, depending on the variable distribution. A p-value less than 0.05 was considered statistically significant.

### 2.4 Feature selection

Feature selection is a vital aspect of ML as it can reduce dimensionality, increase model interpretability, enhance prediction performance and reduce training time. To select the optimal predictors for PVD prediction, we included all variables statistically significant in the univariate analysis and those clinically relevant. The RFE method was applied. We systematically explored feature quantities from 1 to the maximum, for each specific feature quantity, we employed random forest classifier to select the optimal feature subset. In order to evaluate the discriminative capability of different feature subsets, six well-known ML classifiers: Decision Tree (DT) (35), Logistic Regression (LR) (36), Random Forest (RF) (37), Support Vector Machine (SVM) (38), Extreme Gradient Boosting (XGBoost) (39), Adaptive Boosting (AdaBoost) (40) which are widely used in medical research, were used along with 5-fold cross-validation. By comparing the AUC values of the six ML models across different feature subsets, the best feature subset was identified for further model training.

### 2.5 Dataset division and data balancing

A common challenge in ML is overfitting, where the model fits the training data too well but performs poorly on new data. To address this issue, we split the dataset into a training set for model development and a test set for evaluation, maintaining an 8:2 ratio while preserving the balance of positive and negative samples.

Because the dataset comes from hospitalized diabetic patients, the patients with PVD is more than that of without PVD. If unadjusted, the minority class could be overwhelmed by the majority class, leading to suboptimal performance as general ML algorithms often struggle with imbalanced datasets, resulting in bias towards the majority class (41, 42). To address this issue, we applied the Synthetic Minority Oversampling Technique (SMOTE) to the training data, generating synthetic samples for the minority class (43, 44).

### 2.6 Model development and evaluation

We developed six ML models using the balanced training set in a pipeline, including LR, DT, RF, SVM, XGBoost and AdaBoost, and use test set for internal validation. A grid search in combination with 10-fold cross-validation was conducted to optimize the hyperparameters of six ML models to achieve the best AUC score. The details of hyperparameter optimization for each model are listed in **Table 1**. Grid searches determined the best hyperparameter value based on a set of given values (5, 10, 45), all other parameters were set to be default. The performance of each model on the test dataset was assessed using metrics such as accuracy, precision, recall, F1-score, G-mean and AUC-ROC (area under curve of sensitivity versus false positive rate) (34). The overall performance of the prediction model was evaluated using AUC-ROC. Through comprehensive evaluation of multiple metrics, the best performing model was selected for further risk prediction and analysis.

**Table 1:** Hyperparameters of machine learning models.

Model	Hyperparameters	Range	Optimal values
**LR**	penalty	[l1, l2]	l1
	C	[0.01, 0.1, 1.0, 10.0, 50.0]	0.1
	solver	liblinear	liblinear
	max_iter	[100, 500, 1000]	100
**DT**	max_depth	[3, 4, 5, 6, 7, 10, 20,None]	7
	min_samples_split	[2, 5, 10]	10
	min_samples_leaf	[1, 2, 3, 4, 5]	5
	criterion	[gini, entropy]	entropy
**RF**	n_estimators	[10, 15, 20,100, 300]	300
	max_depth	[None, 1, 3, 5, 7, 10]	None
	min_samples_split	[2, 3, 5, 7, 10]	2
	min_samples_leaf	[1, 2, 4, 5]	1
**SVM**	C	[0.1, 1, 10]	10
	kernel	[linear,rbf]	rbf
**XGBoost**	learning_rate	[0.01, 0.1]	0.1
	max_depth	[1, 3, 5, 7, 9]	9
	min_child_weight	[1, 3, 5, 6, 8, 10]	1
	gamma	[0, 0.1, 0.2, 0.4, 0.8, 1]	0
	n_estimators	[100, 300]	300
**AdaBoost**	learning_rate	[0.01, 0.1, 1, 10]	1
	n_estimators	[50, 100, 200,300]	300

LR, Logistic regression; DT, Decision Tree; RF, Random Forest; SVM, Support Vector Machine; XGBoost, Extreme Gradient Boosting; Adaboost, Adaptive Boosting.

### 2.7 Feature importance and model interpretation

Traditional ML models are often viewed as ‘black boxes’ due to their inability to transparently reveal how features influence predictions, a limitation impeding their clinical application. To improve the interpretability of ML model results and analyze influencing factor, we evaluated feature importance within the models and utilized the SHAP method for visual analysis. SHAP, grounded in game theory and first proposed by Lloyd Shapley, offers a framework based on additive feature attribution (46). Its basic idea is to explain the model by calculating the marginal contribution of each feature when added to the model. It can be used to explain various black-box models. This method first calculates the contribution value of each feature, which can be positive or negative, and then accumulates the contribution values of all features to obtain the final prediction. Compared to other methods, it has a solid theoretical foundation and can provide both local and global interpretability simultaneously (47). We leveraged SHAP to provide an explanation for the best-performing model, illustrating feature importance rankings and relationship between the features and the outcomes.

## 3 Results

### 3.1 Baseline characteristics

Following the predefined exclusion and inclusion criteria, 3982 patients were include in our study, with 67 variables extracted. The average age of the patients with T2DM was 60.34 ± 13.55 years, Moreover, baseline characteristics such as age, TBA, BUN, LP-a, the prevalence of DPN, diabetic Retinopathy, Diabetic foot, Hypertension, Diabetic Nephropathy, Cardiac insufficiency and Heart failure (CHF) and Atherosclerosis were significantly higher in PVD patients compared to NPVD patients(p<0.001). **Table 2** displays the baseline characteristics and the results of the univariate analysis for these significant indicators.

**Table 2:** Baseline analysis result of 3982 patients with and without PVD in T2DM.

Variable	Total (n=3982)	NPVD (n=917)	PVD (n=3065)	P value
Age (years)	60.34 ± 13.55	51.57 ± 15.44	62.96 ± 11.73	<0.001**
Weight (kg)	63.61 ± 13.31	66.71 ± 15.16	62.68 ± 12.56	<0.001**
Height (m)	163.00 (158.00,169.00)	165.00 (160.00,170.00)	163.00 (158.00,168.00)	<0.001**
BMI (kg/m^2^)	23.15 (21.55,25.22)	23.86 (22.03,26.23)	23.15 (21.48,25.00)	<0.001**
SBP (mmHg)	133.99 ± 19.30	129.98 ± 17.74	135.19 ± 19.58	<0.001**
Blood pressure difference (mmHg)	55.97 ± 15.71	51.54 ± 14.48	57.29 ± 15.82	<0.001**
UPR-24 (mg/24 h)	86.95 (40.00,189.00)	67.00 (36.10,122.45)	86.95 (40.90,215.30)	<0.001**
HbA1c (%)	8.80 (7.30,10.90)	9.30 (7.40,11.30)	8.80 (7.30,10.80)	0.005**
UCr (μmol/L)	6662.50 (4718.75,9710.25)	7754.009 (5360.50,10955.50)	6611.00 (4590.00,9384.50)	<0.001**
Hb (g/L)	130.00 (115.00,142.00)	133.10 (118.00,146.33)	129.00 (114.07,141.00)	<0.001**
P (mmol/L)	1.17 ± 0.23	1.19 ± 0.27	1.16 ± 0.22	0.001**
APOA1 (g/L)	1.28 (1.16,1.40)	1.27 (1.14,1.40)	1.28 (1.16,1.41)	0.001**
CO2-BC (mmol/L)	23.80 (22.10,25.30)	23.60 (21.65,25.10)	23.80 (22.20,25.40)	<0.001**
GSP (mmol/L)	2.37 (2.11,2.72)	2.42 (2.09,2.77)	2.37 (2.12,2.71)	0.013*
TG (mmol/L)	1.45 (1.05,2.07)	1.49 (1.09,2.15)	1.45 (1.03,2.04)	0.011*
TBA (μmol/L)	4.40 (3.00,6.10)	3.90 (2.80,5.60)	4.40 (3.10,6.20)	<0.001**
CysC (mg/L)	0.86 (0.69,1.10)	0.78 (0.64,0.99)	0.88 (0.71,1.14)	<0.001**
GGT (U/L)	25.00 (19.00,36.00)	28.00 (20.00,40.00)	25.00 (19.00,34.00)	<0.001**
ALT (U/L)	19.00 (14.00,29.00)	21.00 (15.00,34.00)	19.00 (13.00,28.00)	<0.001**
GFR (ml/min)	109.00 (72.00,158.00)	129.00 (87.00,179.00)	105.00 (68.00,151.00)	<0.001**
IBili (umol/L)	7.50 (5.30,10.30)	7.70 (5.55,10.90)	7.40 (5.20,10.20)	0.005**
TBili (umol/L)	10.20 (7.50,13.80)	10.60 (7.70,14.60)	10.10 (7.30,13.70)	0.006**
GLO (g/L)	25.80 (22.70,29.40)	25.10 (22.10,28.50)	26.00 (22.95,29.70)	<0.001**
ALB (g/L)	40.00 (36.80,42.70)	40.70 (37.40,43.30)	39.90 (36.60,42.50)	<0.001**
A/G Ratio	1.54 (1.30,1.80)	1.60 (1.36,1.86)	1.53 (1.29,1.78)	<0.001**
LDH (U/L)	159.00 (139.00,183.00)	151.50 (134.50,176.00)	159.00 (140.00,184.00)	<0.001**
ALP (U/L)	75.00 (66.00,87.00)	77.00 (66.00,89.00)	75.00 (66.00,87.00)	<0.001**
AST/ALT	0.95 (0.75,1.25)	0.89 (0.67,1.16)	1.00 (0.77,1.27)	<0.001**
LP-a (mg/L)	58.00 (28.00,117.25)	46.00 (26.00,108.50)	58.00 (29.00,122.00)	<0.001**
BUN (mmol/L)	5.60 (4.40,7.10)	5.10 (4.00,6.50)	5.70 (4.54,7.27)	<0.001**
SCr (μmol/L)	71.00 (57.00,89.00)	65.00 (53.50,81.00)	73.00 (58.00,92.00)	<0.001**
CP (ng/ml)	2.04 (1.43,2.76)	1.97 (1.32,2.66)	2.04 (1.46,2.79)	0.003**
PT (%)	102.90 (99.40,105.70)	103.40 (100.50,106.20)	102.90 (99.40,105.70)	0.002**
FIB (g/L)	3.54 (2.92,4.42)	3.34 (2.79,4.17)	3.54 (2.94,4.53)	<0.001**
MA (mg/L)	20.40 (8.20,76.23)	15.70 (7.70,47.40)	20.40 (8.30,89.45)	<0.001**
UMACR	25.90 (10.30,113.33)	18.00 (9.00,56.80)	26.20 (10.80,139.10)	<0.001**
DPN	3494 (87.75)	682 (74.37)	2812 (91.75)	<0.001**
DR	574 (14.41)	100 (10.91)	474 (15.46)	0.001**
DF	607 (15.24)	89 (9.71)	518 (16.90)	<0.001**
Hypertension	1931 (48.49)	278 (30.32)	1653 (53.93)	<0.001**
Fatty liver	1346 (33.80)	341 (37.19)	1005 (32.79)	0.014*
DN	1361 (34.18)	243 (26.50)	1118 (36.48)	<0.001**
CHF	893 (22.43)	116 (12.65)	777 (25.35)	<0.001**
DK	497 (12.48)	225 (24.54)	272 (8.87)	<0.001**
Acidosis	189 (4.75)	96 (10.47)	93 (3.03)	<0.001**
Atherosclerosis	409 (10.27)	57 (6.22)	352 (11.48)	<0.001**
Hypertensive heart disease	557 (13.99)	55 (6.00)	502 (16.38)	<0.001**

*, p value<0.05; **,p value<0.01. BMI, body mass index; SBP, systolic blood pressure; UPR-24,24-hour Urine Protein; HbA1c, glycated hemoglobin; UCr, Urinary Creatinine; Hb, hemoglobin; P, Inorganic Phosphate; APOA1, Apolipoprotein A1; CO2-BC, Carbon Dioxide Binding Capacity; GSP, Glycated Serum Protein; TG, triglycerides; TBA, Total Bile Acids, CysC, Cystatin; GGT, Gamma-Glutamyl Transpeptidase; ALT, Alanine Aminotransferase; GFR, Glomerular Filtration Rate; IBili, Indirect Bilirubin; TBili, Total Bilirubin; GLO, Globulin; ALB, Albumin; A/G Ratio, Albumin/Globulin Ratio; LDH, Lactate Dehydrogenase; ALP, Alkaline Phosphatase; LP-a, lipoprotein (a); BUN, blood urea nitrogen; SCr, serum creatinine; CP, C-peptide; PT, Prothrombin Activity; FIB, Fibrinogen; MA, Microalbuminuria Testing; DPN, Peripheral Neuropathy desease; DR, diabetic Retinopathy; DF, Diabetic foot; DN, Diabetic Nephropathy; CHF, Cardiac insufficiency and Heart failure; DK, Diabetic Ketos.

### 3.2 Feature selection

**Figure 2:**
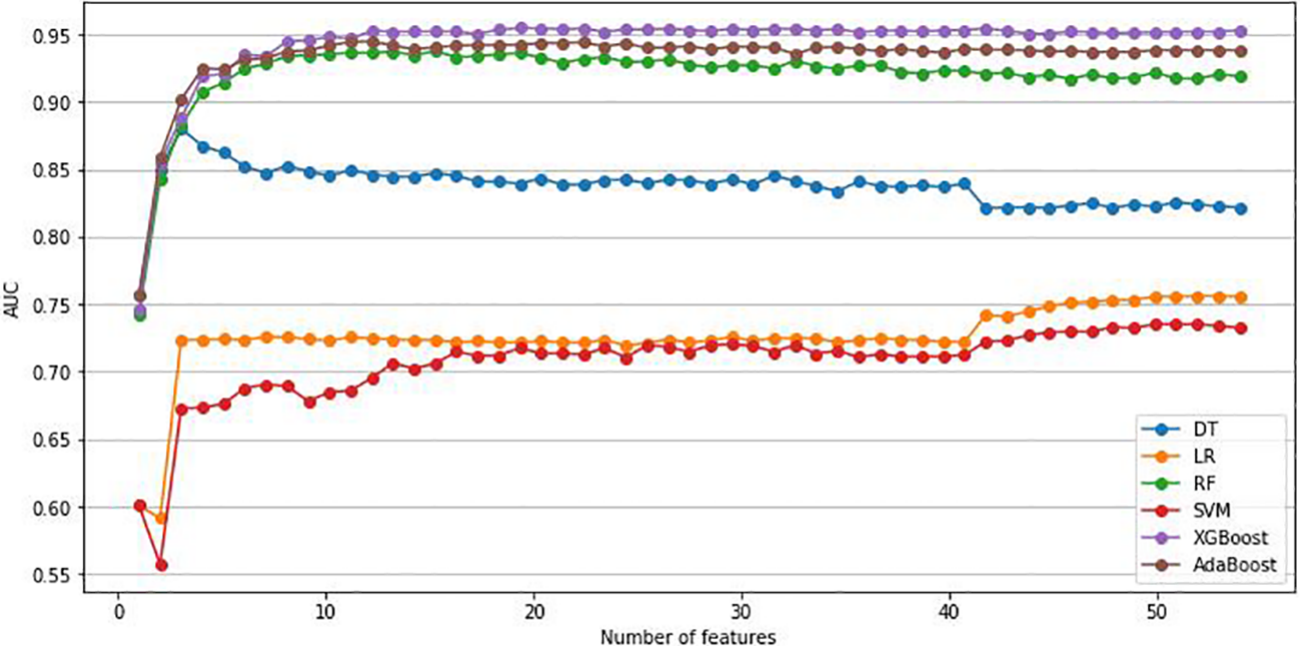
Change of AUC based on the number of features. AUC, area under the curve; LR, Logistic regression; DT, Decision Tree; RF, Random Forest; SVM, Support Vector Machine; XGBoost, Extreme Gradient Boosting; Adaboost, Adaptive Boosting.

As previously mentioned, this study, utilized the RFE method and six ML algorithms to identify the optimal number of features that have the best prediction performance. We included 53 variables in the RFE method, comprising 47 variables identified as significant in univariate analysis and 6 clinically relevant variables. **Figure 2** illustrates the variation in AUC based on the number of features in each classification algorithm. According to the result,12 variables were selected to build the prediction models: age, Hb, TBA, LP-a, 24-hour urine protein (UPR-24), GGT, glycated serum protein (GSP), BMI, Lactate Dehydrogenase (LDH), ALP, apolipoprotein A1 (APOA1) and BUN.

### 3.3 Modeling and evaluation

**Figure 3:**
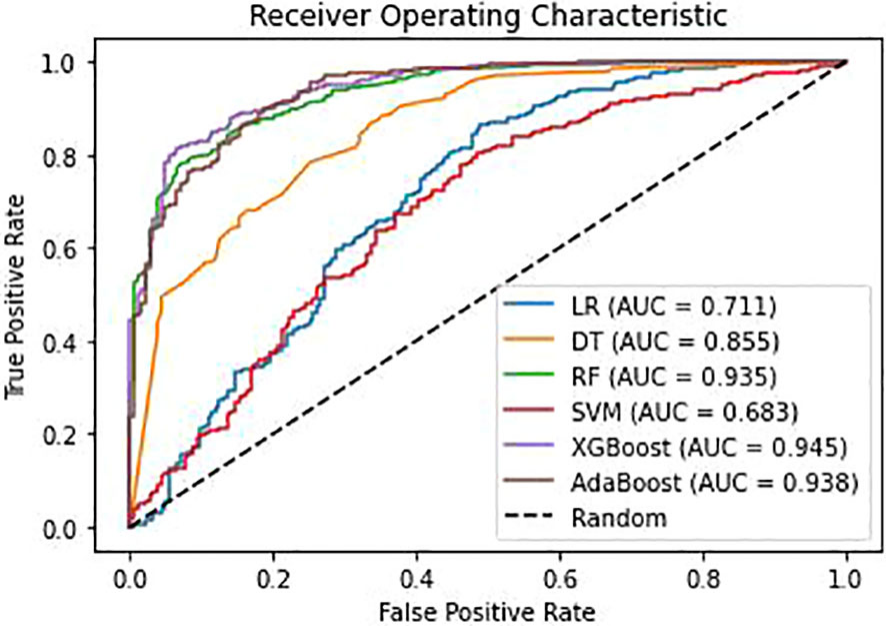
Receiver operating characteristic(ROC) curves for different models. AUC, area under the curve; LR, Logistic regression; DT, Decision Tree; RF, Random Forest; SVM, Support Vector Machine; XGBoost, Extreme Gradient Boosting; Adaboost, Adaptive Boosting.

The training set comprised 2452 positive samples and 733 negative samples before balancing. After oversampling with the SMOTE algorithm, the number of negative samples also increased to 2452. The validation set included 613 positive samples and 184 negative samples. Models including DT, LR, RF, SVM, XGBoost and AdaBoost were developed using the training set with the above-mentioned 12 variables. The performance assessment results, including accuracy, precision, recall, F1-score, G-mean, and ROC-AUC, are detailed in **Table 3**; **Figure 3**. The results showed that in the validation set, XGBoost achieved the highest AUC (0.945), accuracy (0.890), recall (0.927), and F1-value (0.928). Adaboost exhibited the highest precision (0.943), G-mean (0.853). Overall, the performance of the XGBoost and Adaboost were significantly superior to the other four models, and XGBoost is the best model. Thus, XGBoost was selected for further prediction and analysis.

**Table 3:** Performance comparison of different machine learning models.

Model	AUC	G-mean	Accuracy	Precision	Recall	F1-score
**LR**	0.711	0.651	0.666	0.858	0.679	0.758
**DT**	0.855	0.750	0.794	0.896	0.828	0.861
**RF**	0.935	0.817	0.873	0.918	0.917	0.918
**SVM**	0.683	0.641	0.706	0.846	0.755	0.798
**XGBoost**	**0.945**	0.843	**0.890**	0.930	**0.927**	**0.928**
**Adaboost**	0.938	**0.853**	0.871	**0.943**	0.886	0.913

The best results are in bold. AUC, area under the curve; LR, Logistic regression; DT, Decision Tree; RF, Random Forest; SVM, Support Vector Machine; XGBoost, Extreme Gradient Boosting; Adaboost, Adaptive Boosting.

### 3.4 Feature importance and SHAP values

**Figure 4:**
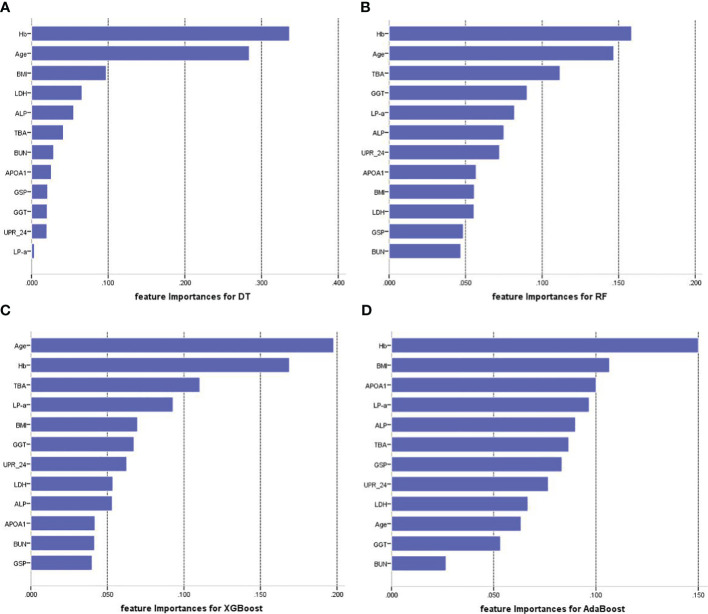
Feature importance of each of the features for four prediction models. **(A)** Feature importance for DT. **(B)** Feature importance for RF. **(C)** Feature importance for XGBoost. **(D)** Feature importance for AdaBoost. Hb, hemoglobin; TBA, total bile acids; LP-a, lipoprotein(a); UPR-24, 24-hour urine protein; GGT, gamma-glutamyl transpeptidase; GSP, glycated serum protein; BMI, body mass index; LDH, Lactate Dehydrogenase; ALP, Alkaline Phosphatase; APOA1, apolipoprotein A1; BUN, blood urea nitrogen.

**Figure 5:**
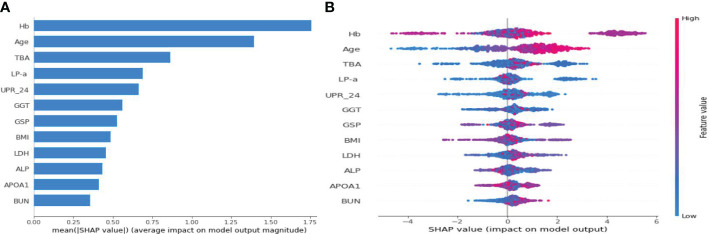
SHAP summary plot for the 12 clinical features contributing to the XGBoost model for the test dataset. **(A)** SHAP feature importance measured as the mean absolute Shapley values. **(B)** The distribution of the impact of a risk factor value on the model output.The contribution of each feature of each patient to the model corresponds to a dot. The dots are coloured according to the values of features. Red represents a higher feature value, and blue represents a lower feature value. SHAP, Shapley Additive Explanation.

**Figure 4** illustrates the feature importance rankings for four ML models. The rankings are not quite the same. Key factors impacting PVD were identified as age, Hb, TBA and LP-a. Since XGBoost was the optimal model for predicting PVD, we used SHAP to elucidate the relationship between the features and the output of the XGBoost model. **Figure 5A** demonstrates the average effect of each risk factor on the magnitude of the model output. As can be seen, Hb, age, TBA, LP-a and UPR-24 have large contribution to the model output, while factors such as LDH, ALP, APOA1 and BUN have a small impact on the model output. **Figure 5B** offers an insight into the positive or negative effects of these factors on the XGBoost model. The summary SHAP values of the risk factors in **Figure 5B** are shown in descending order in a top-down view, with the most influential risk factors at the top. Higher SHAP values suggest an increased likelihood of PVD occurrence. The red dots represent higher feature values, and the blue dots represent lower feature values. This analysis identifies Hb as the most critical factor in PVD, with its relationship to PVD risk being complex and non-linear. Age ranks as the second most influential factor, exhibiting a strong positive correlation with the risk of PVD.

### 3.5 Model application for individual

**Figure 6:**
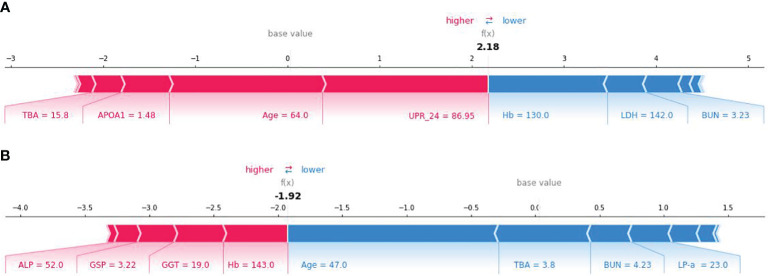
SHAP force plot for explaining of individual’s prediction results. **(A)** SHAP force plot for a PVD patient; **(B)** SHAP force plot for a NPVD patient.

SHAP method can also be used to analyze the influencing factors of an individual developing PVD (**Figure 6**). Here, red area indicates that the feature increases the probability of PVD, while the blue indicates the feature decreases the probability of PVD. The wider the width of the color region, the greater the impact of the feature on PVD. The value f(x) represents the cumulative SHAP value for each patient. The base value is the average SHAP value of all patients. The upper panel demonstrates accurate prediction of a PVD patient. The reasons for predicting it as PVD are older age and larger TBA among others. Conversely, The panel below shows correct identification of a non-PVD patient. The reasons for predicting it as NPVD are younger age, lower levels of TBA, and normal BUN level. The XGBoost model can effectively distinguish between PVD and NPVD patients, providing tailored risk assessments for each individual.

## 4 Discussion

PVD is a prevalent macrovascular complications of T2DM and is a long-standing complication. It can increase the risk of cardiovascular complications, and it’s a major contributor to the development of diabetes-related foot disease (DFD), diabetes-related foot ulceration(DFU), and lower-limb amputation (LLA) (48). DFU, LLA are associated with significant disability and increased mortality, with a 5-year mortality risk of 70% after LLA and 50% after DFU (49). Most PVD patients have systemic atherosclerosis, and many die from cardiovascular and cerebrovascular events. PVD is a global health problem that affects over 200 million people worldwide. One reason for high prevalence is that in many cases, PVD is silent and less than 20% of patients report typical symptoms of PVD. Due to peripheral neuropathy, the prevalence of asymptomatic PVD is even higher in diabetic patients, estimated to be almost one-third of all diabetic patients (50). Early symptoms of PVD are non-specific,. Once there is resting pain, intermittent claudication, ischemic gangrene, etc., trauma treatment or even amputation is needed, which will cause significant psychological and physiological harm to the patient (51). Therefore, prompt diagnosis and treatment of PVD in DM patients are crucial. Currently, PVD diagnosis primarily depends on imaging techniques like B-ultrasound and MRI (52). However, these methods can be costly and time-consuming, and with the rising number of diabetes cases, imaging every diabetic patient presents a considerable challenge. Therefore, developing a risk prediction model to identify high-risk individuals for targeted imaging is essential.

Several studies have attempted to build risk predictive models for PVD. Schallmoser et al. (4) developed ML models to predict the risk of developing PVD and five other complications in individuals with diabetes and prediabetes simultaneously using electronic health records from Israel, However, due to the non-specificity of the indicators used for all six complications and an unaddressed imbalanced dataset, the models showed relatively poor predictive performance despite a large data size. Liang et al. (52) developed a nomograph to predict the risk of PAD, however, nomograph method is inapplicable for large-scale datasets. Wilcox et al. (53) and Majid Khan et al. (50) only employed statistical description and univariate analysis methods to analyze factors influencing PVD. To date, no study has specifically used machine learning to establish a PVD risk prediction model. This study aims to develop such a model for T2DM patients in the Hainan region, using six machine learning models based on electronic medical records. The XGBoost model exhibited the highest predictive performance with the highest AUC of 0.945 and recall of 0.927 on the testing dataset, the high AUC and high recall rate of the model shows that it has good reliability. Given that the strength of the associations found can vary across different ML models, we compared the feature importance rankings of four different models and found that most models considered Age, Hb, LP-a to be the most important influencing factors, which makes the influencing factors we have identified more convincing.

The developed model has several advantages. Firstly, RFE with 5-fold cross-validation was used to select the best features subset for developing prediction model. Secondly, to mitigate the bias towards the majority class in unbalanced datasets and prevent overfitting, we utilized the SMOTE for oversampling. Thirdly, we developed six classical ML prediction models and determined that the XGBoost model was the best after comparing their performance. Fourthly, we employed the SHAP method to explain the relationship between the input features and the output of the XGBoost model, thereby facilitating an intuitive understanding for clinicians and enhancing the model’s clinical applicability. Additionally, we conducted an analysis of the factors influencing individual PVD progression using the SHAP method, which can assist clinical doctors in personalized preventive treatments.

In this study, the prevalence of PVD in patients with T2DM was 77%, significantly higher than previously reported figures. This elevated prevalence could be attributed to multiple factors. Firstly, the actual prevalence of PVD may be greatly underestimated due to asymptomatic early stages leading to undiagnosed cases (48, 54). Secondly, our study population consisted of hospitalized patients with T2DM, who likely had more severe conditions and a higher prevalence of complications. Thirdly, the data source, a leading comprehensive hospital in Hainan province, typically admits patients with relatively serious conditions. Fourthly, the mean age of our study subjects is relatively high at 60.34 years, and age is an important risk factor for PVD.

The XGBoost model and three other models all identified Hb as the most important risk factor for developing PVD in patients with T2DM. And the SHAP values plot indicate that the influence of Hb on PVD is complex and non-linear. Decreased Hb levels can lead to inadequate oxygen supply, particularly in already damaged blood vessels, which can exacerbate vascular damage and inflammatory responses, thus increasing the risk of cardiovascular events and lower limb arterial disease, Additionally, Hb is an important indicator for diagnosing anemia, and anemia can also impact diabetes control and the progression of complications (52). Individuals with both diabetes and anemia suffered more comorbidities, were more likely to be hospitalized and have a higher risk of death (55). Therefore, monitoring and managing Hb levels are crucial in preventing and treating PVD in diabetic patients. Further research is still needed to explore the exact mechanisms and interrelationships between hemoglobin and PVD in diabetes.

Age is recognized as the second influencing factor in the development of PVD. As age increases, the risk of this disease increases accordingly. This relationship is well-documented in medical literature (4, 17, 31, 56). This is due to the fact that over time, they are more prone to developing atherosclerosis, declining vascular function, and complications from other chronic diseases. The high blood sugar levels in diabetic patients accelerate the progression of atherosclerosis and make blood vessels more susceptible to damage. Furthermore, aging leads to reduced vascular elasticity and endothelial dysfunction, which are further worsened by hyperglycemia. Additionally, As people with diabetes age, they may also face other age-related diseases and health problems, such as hypertension, hyperlipidemia and obesity, which may be closely associated with PVD. Therefore, it is crucial for older diabetic patients to prioritize blood sugar control and vascular protection. Regular monitoring of blood sugar, blood pressure, and lipid levels, along with lifestyle improvements such as a balanced diet, moderate exercise, and tobacco and alcohol cessation, and adherence to medication as prescribed by their healthcare professional, can help reduce the risk of PVD in diabetic patients. Regular check-ups and close collaboration with healthcare professionals are also vital.

Lipoprotein-a (LP-a) is a genetically determined, cholesterol-rich plasma lipoprotein. Lp-a has proinflammatory and proatherogenic properties. Its serum level is associated with atherosclerotic cardiovascular diseases, including PAD. Previous studies have shown that Lp-a is a significant independent risk factor for PVD and is also associated with more severe forms of PVD in specific populations (57–59). Our study also indicated that LP-a is an important risk factor of PVD in T2DM, which is consistent with the aforementioned studies.

Our model indicated that TBA was an important factor for PVD. Patients with higher TBA are more likely to develop PVD. There are several possible mechanisms for this. First one is elevated serum TBA levels can damage vascular endothelial cells, thereby participating in the mechanism of atherosclerosis through endothelial injury (60). Another research suggests that TBA act as inhibitors of 11 β-hydroxysteroid dehydrogenase. Elevated TBA levels may inhibit the activity of 11 β-hydroxysteroid dehydrogenase, resulting in increased levels of cortisol in the body. Cortisol can bind to mineralocorticoid receptors and produce aldosterone-like effects, leading to an increase in circulating blood volume and affecting blood pressure. This process is believed to contributes to the occurrence and progression of atherosclerosis (61). More research is needed to further clarify the mechanism by which TBA are involved in the development of PVD.

Other risk factors for PVD also warrant consideration, though they may not be ranked as high as the above four factors or might not be included in our machine learning prediction model. Analyses in America have shown that individuals with PVD typically have a slightly lower average BMI. This could be attributed to smoking, a significant risk factor for PVD, which can also lead to weight loss (14). A retrospectively study indicated that BUN was an independent risk factor for PVD in patients with T2DM. BUN is an easily recognizable, widely accessible, and inexpensive marker that can identify patients at high risk for PVD (52). Our study obtained similar results concerning the aforementioned factors.

To the best of our knowledge, this study is the first to apply various ML algorithms to establish prediction models for PVD in T2DM and analyze its risk factors. Nonetheless, this study has limitations. Firstly, only participants with diabetes were included, and therefore solely diabetes-related indicators were explored. If a control group of healthy individuals had been integrated, the prediction outcomes could have varied. Secondly, the current research was a single-center retrospective modeling study with only an internal validation. The applicability of our results to other regions remains to be verified. Thirdly, imputation and feature selection were conducted prior to the training and validation sets split, which may have impacted the final models. Finally, future research needs to address the lack of collected and analyzed data on patients’ subjective descriptions, such as their duration of diabetes and smoking habits, which have been reported as associated with PVD in T2DM (14).

## 5 Conclusions

The rapidly increasing prevalence of T2DM has attracted international attention. As a long-standing complication, PVD imposes a significant burden on individuals and society. Identifying high-risk individuals early is crucial for preventing this complication. In our study, we developed ML models to identify individuals with T2DM at high risk of developing PVD and analyzed the risk factor for PVD. The SHAP method enhanced the interpretability of our ML models, aiding clinicians in understanding the models’ rationale and improving the practical applicability of the prediction models in clinical settings. Our team is committed to refining these PVD prediction models in future studies by addressing the current limitations.

## Data availability statement

The original contributions presented in the study are included in the article/supplementary material . Further inquiries can be directed to the corresponding author.

## Ethics statement

The studies involving humans were approved by ethics committee of Hainan Medical University (No.: HYLL-2021-388). The studies were conducted in accordance with the local legislation and institutional requirements. Since this is a study strictly based on de-identified hospital information system data, individual informed consent is not required.

## Author contributions

LL: Formal analysis, Funding acquisition, Methodology, Software, Validation, Visualization, Writing – original draft, Writing – review & editing, Conceptualization, Investigation, Project administration, Supervision. BB: Data curation, Writing – review & editing. LC: Funding acquisition, Writing – review & editing. MG: Writing – review & editing, Funding acquisition. FJ: Conceptualization, Writing – review & editing.
